# How lipids affect the energetics of co-translational alpha helical membrane protein folding

**DOI:** 10.1042/BST20201063

**Published:** 2022-02-25

**Authors:** Ryan Brady, Nicola J. Harris, Grant A. Pellowe, Samuel Gulaidi Breen, Paula J. Booth

**Affiliations:** 1 King's College London, Department of Chemistry, Britannia House, 7 Trinity Street, London SE1 1DB, U.K.; 2 The Francis Crick Institute, 1 Midland Road, London NW1 1AT, U.K.

**Keywords:** co-translational folding, lipids, membrane proteins, protein folding

## Abstract

Membrane proteins need to fold with precision in order to function correctly, with misfolding potentially leading to disease. The proteins reside within a hydrophobic lipid membrane and must insert into the membrane and fold correctly, generally whilst they are being translated by the ribosome. Favourable and unfavourable free energy contributions are present throughout each stage of insertion and folding. The unfavourable energy cost of transferring peptide bonds into the hydrophobic membrane interior is compensated for by the favourable hydrophobic effect of partitioning a hydrophobic transmembrane alpha-helix into the membrane. Native membranes are composed of many different types of lipids, but how these different lipids influence folding and the associated free energies is not well understood. Altering the lipids in the bilayer is known to affect the probability of transmembrane helix insertion into the membrane, and lipids also affect protein stability and can promote successful folding. This review will summarise the free energy contributions associated with insertion and folding of alpha helical membrane proteins, as well as how lipids can make these processes more or less favourable. We will also discuss the implications of this work for the free energy landscape during the co-translational folding of alpha helical membrane proteins.

## Introduction

Membrane proteins reside in a chemically complex environment. They must interact with the hydrophobic bilayer interior and the aqueous environment outside the bilayer, as well as the interface between the two. The final folded protein structure is crucial for correct protein activity, and for membrane proteins the hydrophobic bilayer is involved in the formation of this correct structure. This makes folding a complex process, with misfolding known to cause trafficking problems and also lead to disease [1]. Although the primary sequence of the protein dictates the final structure of a protein, as described by Anfinsen in 1973 [2], this has been shown to be an oversimplification. Folding is affected by many factors as well as the environment and folding can require assistance by chaperone proteins to prevent protein aggregation [3,4].

Folding is key to cellular life, and it is important to understand the molecular basis and driving forces behind this fundamental process. Membrane proteins are of high physiological importance and are significant drug targets. Their complex folding leads to problems expressing membrane proteins *in vitro*, making them more difficult to study. Folding studies have investigated the dependence of structure formation on the lipid environment [5–10] using full-length protein that has been expressed in cells and purified. Such work has been necessary to probe the mechanistic details including thermodynamics and how the lipid bilayer influences transmembrane (TM) helix insertion and protein folding. *In vivo* however, most alpha helical membrane proteins fold during chain elongation from the N- to C- terminus as they are translated by the ribosome, and they also insert into the membrane co-translationally [11–13]. This means that early helices can form tertiary interactions with each other in the bilayer while later helices are still being translated by the ribosome. Co-translational folding of alpha-helical proteins *in vivo* is assisted by membrane targeting and insertion apparatus. In *E. coli,* growing nascent chains are delivered to the SecYEG translocon by the signal recognition particle (SRP), and the ribosome continues to translate the elongating nascent chain while the translocon assists insertion of the TM helices across the bilayer [13].

**Figure 1.**
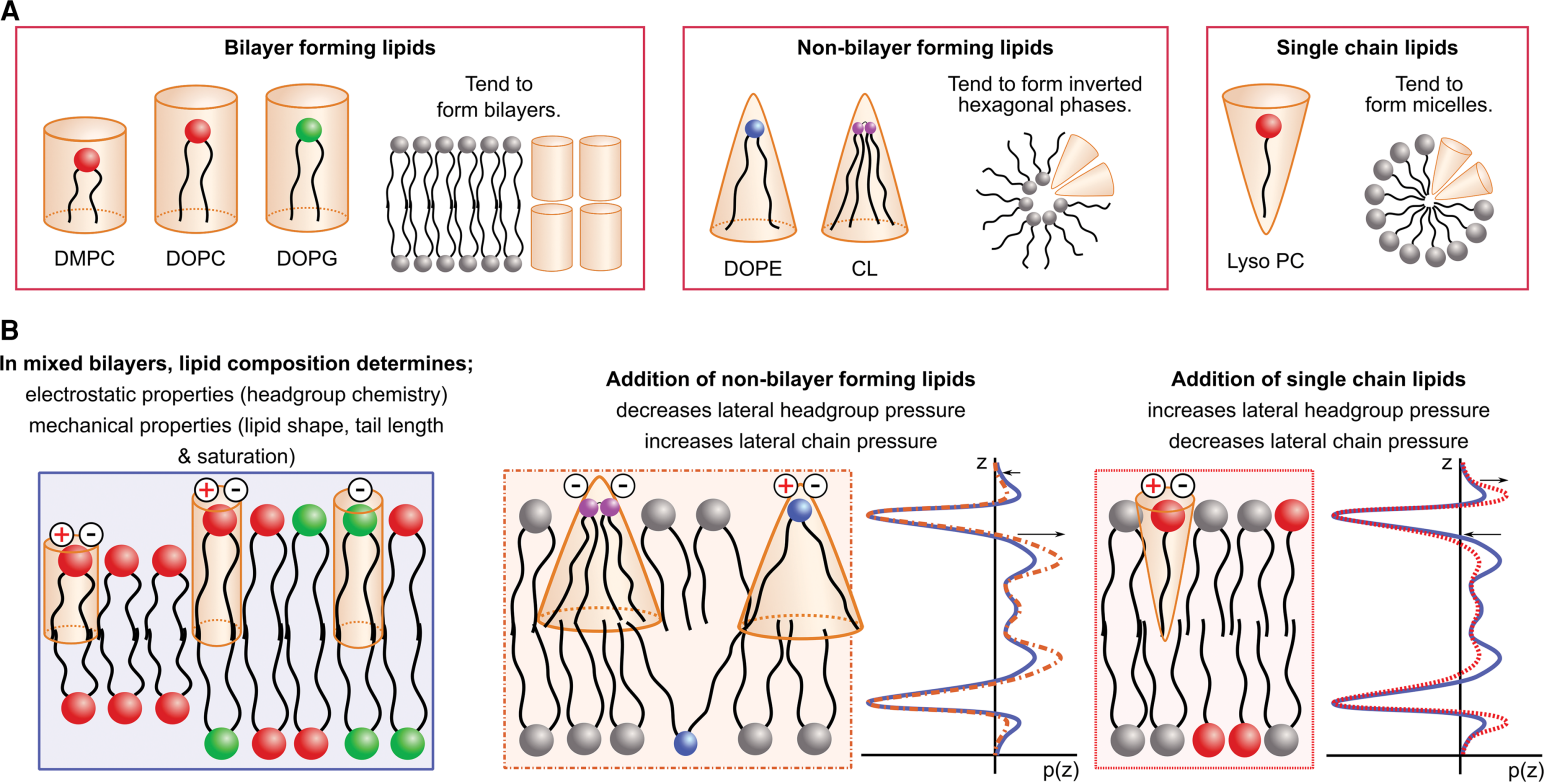
Lipid composition determines bilayer properties. (**A**) Examples of lipids commonly used in membrane protein folding studies. Lipids such as those with PC headgroups tend to form bilayers, while non-bilayer forming lipids such as DOPE and cardiolipin (CL) adopt hexagonal phases. Single-chain lipids such as Lyso PC tend to form micelles. (**B**) Lipid composition determines the overall electrostatic and mechanical properties of mixed lipid bilayers. Introducing non-bilayer forming lipids alters the lateral pressure profile of a bilayer.

Once the extending polypeptide emerges from the ribosome, all stages of membrane protein folding are affected by the lipid composition of the bilayer — association with the bilayer [9,14,15], insertion into the bilayer [15–25] and folding in the bilayer [5–7,9]. The translocon seems to be affected by the lipid composition, with its stability and function in *E. coli* shown to depend on the presence of cardiolipin [26]. There are thousands of different biological lipids with many different properties that can alter the properties of the bilayer (Figure 1), and in turn affect folding. Lipids can have saturated or unsaturated chains and symmetric or asymmetric chains. They can have different types of headgroups, which can be charged or neutral. Lipid headgroups also have different hydrogen-bonding capabilities [27], for example the ethanolamine headgroup in phosphatidylethanolamine (PE) lipids is able to form both intra- and inter-headgroup hydrogen bonds [28]. Bilayers can have a mixture of bilayer-forming and non-bilayer forming lipids, and these alter the lateral pressure profile across the membrane (Figure 1B). Cell membranes are composed of a mix of all these different types of lipids, altering the fluidity and thickness of the bilayer. The inner membrane of gram-negative bacteria such as *E. coli* contains mostly PE with phosphatidylglycerol (PG) and cardiolipin, while the cytoplasmic membrane of gram-positive bacteria contains mainly PG lipids and branched acyl chains [29]. Prokaryotes are able to alter their bilayer composition in response to things such as temperature, pH and pressure [30,31]. Different organelles in eukaryotes have different lipid compositions leading to membrane proteins experiencing different lipid environments during different stages of trafficking [10]. For example, the plasma membrane contains mostly phosphatidylcholine (PC) lipids and cholesterol, whereas the endoplasmic reticulum contains very little cholesterol but contains phosphatidylinositol [10]. Although we know the lipid compositions of various organisms and organelles, it is not well understood precisely what intermolecular forces and interactions underpin the bilayer influence on protein folding and stability.

**Figure 2.**
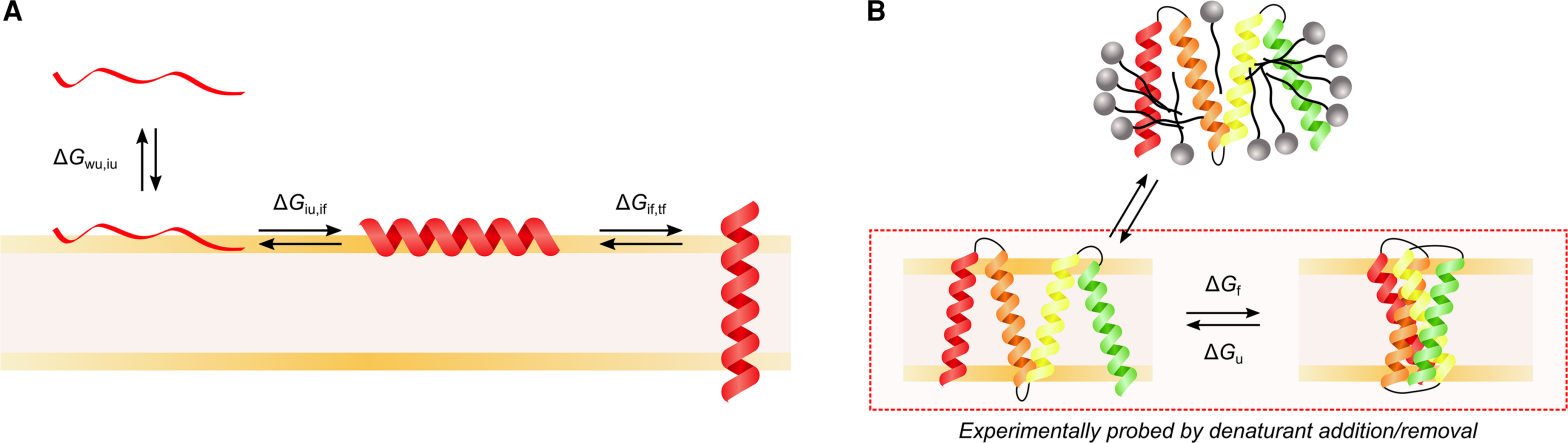
Different methods to measure the free energy landscape of folding. (**A**) A computational model for the insertion of a hydrophobic peptide into a lipid bilayer [34]. An initially unstructured peptide in water partitions to the bilayer interface, where it folds to adopt an alpha-helical structure and inserts across the bilayer. ΔG values denote standard transfer free energies, with subscripts indicating states involved. Each state is described by two letters, with the first denoting environment (*w*, water; *i*, interface; *t*, transmembrane) and the second denoting the state of the peptide (*u*, unfolded; *f*, folded). For co-translational insertion of membrane proteins *in vitro* and *in vivo*, TM domains form alpha-helices in the ribosome exit tunnel rather than folding interfacially. (**B**) Measuring the folding free energy of a full-length membrane protein with equilibrium folding/unfolding. The ΔG of folding (ΔG_f_) and unfolding (ΔG_u_) are determined by measuring the change in structure upon addition and removal of a denaturant (shown in the red dashed box). Alternatively, a protein can be unfolded in detergent with either SDS or urea, and then refolded into a lipid bilayer.

**Figure 3.**
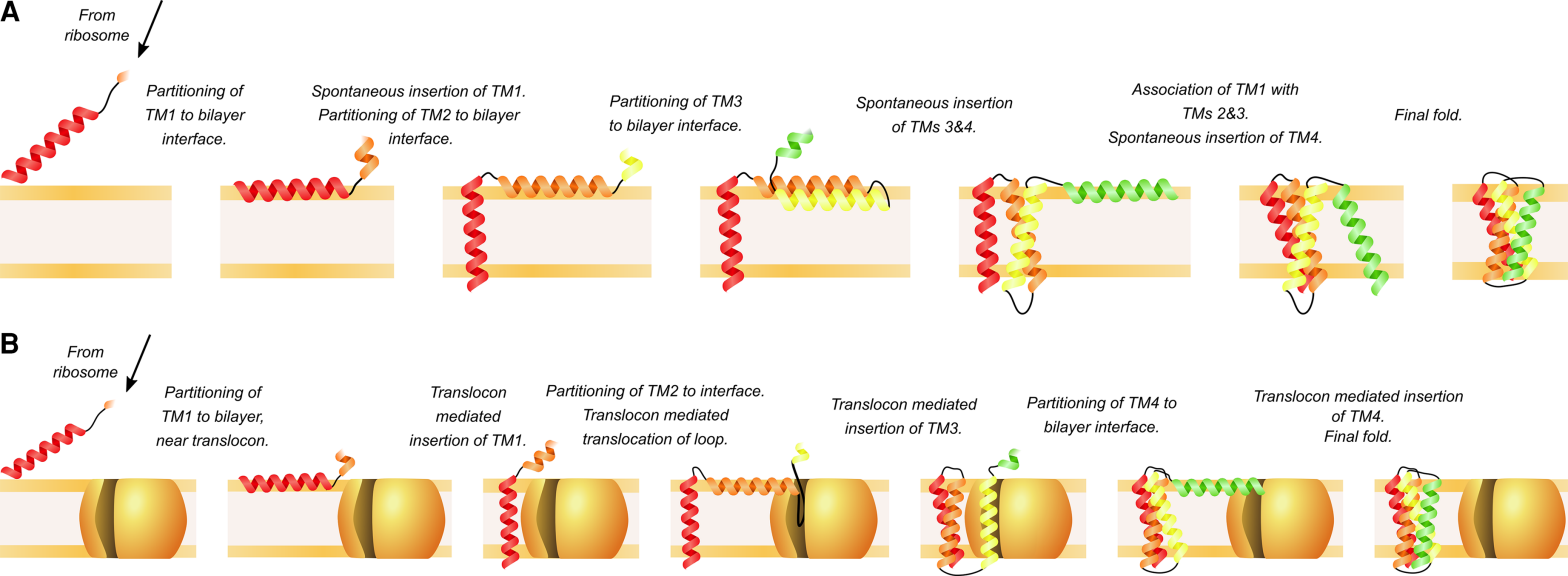
The stages involved in co-translational folding. The steps involved in co-translational TM interfacial headgroup partitioning, bilayer insertion and folding either without (**A**) or with (**B**) the translocon. Folding begins while later TMs are being translated and inserted in the bilayer. Some of these stages, and free energies associated with each stage, are the same as those measured using *in vitro* methods (as illustrated in Figure 2). We use the models in this figure to highlight the similarities and differences in pathways between translocon-assisted and spontaneous TM insertion and membrane protein folding, and to show that interactions between the nascent chain and the lipid bilayer are involved in all stages.

During folding there are free energy contributions from solvation in the cytoplasm vs. in the membrane, association with the headgroup region of the lipids, inserting/partitioning of a TM helix across the bilayer, and folding once within the bilayer (Figure 2A,B). These free energies all contribute to the overall free energy landscape of membrane insertion and folding, and negative free energy contributions must balance against positive free energy contributions in order for folding to be successful. The free energy landscape includes enthalpic and entropic contributions, and measurements of thermodynamics and kinetics are used to define what the different contributions are at each stage of folding. During co-translational folding, the free energy landscape is likely to change with each amino acid translated and is challenging to measure both *in vivo* and *in vitro*. Thermodynamic and kinetic work therefore began on simpler systems such as peptides, and *in vitro* folding studies typically used fully translated proteins which had been unfolded in denaturant and refolded, with the change in structure and the kinetics between the two conformations measured (Figure 2B). A key advance for alpha-helical membrane protein folding in 1990 was the ‘two-stage model' [32,33], in which TM helices were deemed to be independently stable entities and insert into the bilayer in the first stage. In the second stage, these helices interact with each other in the bilayer to form the final folded structure. The study of the insertion of individual peptides is similar to the first stage, while some aspects of *in vitro* denaturant studies address stage two. However, this two-stage model is oversimplistic as not all helices are independently stable [34–37], and at the time there was limited detailed knowledge of co-translational insertion *in vivo* (as illustrated in Figure 3). Previous reviews have discussed in depth the energetics and mechanisms of membrane protein folding [34,38], and also the effect of lipids on folding [10]. This review will summarise the effects of lipids on the free energy landscape of alpha-helical membrane protein insertion and folding, using peptides and denaturant studies of full-length proteins. We will then discuss more recent work on co-translational folding, and the possible effects of the lipids on the co-translational folding free energy landscape.

## Peptides can be used as a simple model for TM association and insertion into the bilayer

Although the translocon is present *in vivo* to aid insertion and folding, when no translocon is present *in vitro* TM peptides and nascent alpha-helical membrane proteins can partition into the bilayer spontaneously [16–18,22,24,39–42] driven predominantly by favourable interactions between hydrophobic amino acid side chains and the hydrocarbon rich bilayer core. This has been taken advantage of in the use of peptides to investigate the thermodynamics governing TM integration into the bilayer (Figure 2A). Work on peptides has shown that the free energy associated with partitioning peptide bonds into the membrane interface and the bilayer core is unfavourable, and this is markedly reduced by hydrogen-bonding of the peptide backbone into an alpha-helical secondary structure [43,44]. Insertion of an unstructured peptide into the bilayer core is so energetically disfavoured that TM helices are only ever observed to insert across the membrane (or indeed exist within the membrane) when alpha-helical [34]. This also means that *in vitro* folding studies which measure partially unfolded states of full-length proteins are still biologically relevant, as TM helices are not expected to be fully denatured when associated with a bilayer. Even when in an alpha-helical structure, there is still a substantial free energy cost associated with the insertion of peptide bonds into the hydrophobic bilayer interior. In order to be stably inserted across a bilayer, TM domains must be rich in amino acids with hydrophobic side chains [45–47], which have favourable free energies of insertion that outweigh the energetic cost of peptide bond dehydration. Polar residues are rarely present at sites in TM helices facing the hydrocarbon bilayer core, and those which are located in such positions tend to be essential for protein function.

From work using peptides, hydrophobicity scales for TM insertion have been developed [47–49]. These scales have led to the development of prediction tools for the free energy of insertion of TM sequences [37,50]. Peptide work has also enabled the development of a biological hydrophobicity scale [51], and the position dependent contribution of each amino acid on insertion efficiency through the translocon has also been quantified [37]. It is therefore now possible to find whether there are any TM helices in a protein that are less likely to insert across a bilayer based on sequence alone.

While easier to work with than full-length membrane proteins, measuring the free energy of association and insertion of peptides experimentally is not trivial, as their hydrophobicity makes them prone to aggregation when not associated with a bilayer*.* Peptides are, however, particularly amenable to all-atom molecular dynamics (MD) simulations, which have proven to be a powerful tool to study the dynamic processes associated with peptide adsorption, folding, and bilayer insertion [52,53]. Recent advances in hardware and software have pushed the achievable timescales of MD simulations to the multi-microsecond range, but the processes involved in peptide insertion can take hours to observe experimentally [53]. In the specific case of membrane active peptides, it has been rigorously validated that the free energy of bilayer insertion is temperature independent over a wide range (as discussed in detail in [52]). As such, it is possible to quantitatively estimate the free energy of bilayer partitioning from high-temperature molecular dynamics (HT-MD) simulations, where transitions are accelerated. Ensemble average properties (e.g. free energies, average peptide orientation) derived from HT-MD have been shown to be in excellent agreement with those measured experimentally [52,53]. HT-MD has advanced our understanding of the molecular details of peptide insertion, as well as the properties which drive insertion such as peptide length and hydrophobicity [52]. Recent combined experimental-computational [41,42] studies have been used to show that the binding and insertion of certain small peptides is strongly dependent on the composition of the lipid bilayer. It was found that the peptides studied interacted differently with 1-palmitoyl-2-oleoyl-glycero-3-phosphocholine (POPC) and 1-palmitoyl-2-oleoyl-sn-glycero-3-phospho-(1′-rac-glycerol) (POPG) membranes and behaved differently in gel and fluid membranes. In some cases the peptides studied bound to the headgroups of either POPC or POPG membranes, but only folded into a helix in POPG (Table 1).

**Table 1:** Selected examples of how lipids affect the free energy landscape of insertion, folding and stability of peptides and alpha-helical membrane proteins

Protein	System	Qualitative effect of lipid on insertion/folding	Free energy landscape: Effect on TM-headgroup association	Free energy landscape: Effect on TM insertion	Free energy landscape: Effect on folding and stability once in bilayer	Ref
Small cationic antimicrobial peptide HSP1	Computational and experimental — binding and folding of peptide into lipid headgroups	Folds to helix in POPG but not POPC, but does bind to both Prefers fluid to rigid bilayers	TM association not affected by headgroup, but does affect folding into helix Rigid bilayers disfavour peptide binding and folding			[42]
Bacteriorhodopsin (bR)	Refolding into liposomes from SDS denatured state	DPoPE decreased folding when added to DPoPC LysoPPC increased folding when added to DPoPC		Less stored curvature stress increases amount of protein that inserts into bilayer	Less stored curvature stress increases the folding rate Protein in high curvature stress bilayer is more stable	[9]
Diacylglycerol kinase (DGK)	Refolding into liposomes from urea-denatured state	Rate and yield of folding increases when amount of DOPG increased Rate and yield of folding decreases with addition of DMPC or lysoOPC		Decreased stored curvature stress decreased the folding yield, but DOPG increased it	Decreased stored curvature stress decreased the rate, but DOPG increased it DOPG stabilised the folded state over the unfolded state, by either reducing activation energy of folding or lowering rate of competing misfolding	[7]
LeuT	Urea denaturation in liposomes	Different ratios of DOPC:DOPE and DOPC:DOPG tested. Mechanical and charge properties of the bilayer affect the stability of LeuT against urea			Unfolding free energy decreases when DOPG or DOPE are reduced in DOPC bilayers: ΔG_U_^H2O^ 2.9 kcal mol^−1^ in 50 : 50 DOPC:DOPE decreases to 2.6 kcal mol^−1^ in 80 : 20 DOPC:DOPE 3.8 kcal mol^−1^ in 50 : 50 DOPC : DOPG decreases to 2.5 kcal mol^−1^ in 80 : 20 DOPC : DOPG	[5]
LacY	Reconstitution into liposomes from detergent	Reconstitution efficiency decreases when DOPE is >50%, while thermal stability of LacY increases >50% DOPG increases reconstitution efficiency		DOPE lateral chain pressure decreases likelihood of LacY insertion DOPG increases likelihood of LacY insertion	DOPE improves thermal stability	[6]
LacY	Refolding into liposomes from a urea denatured state	Refolding efficiency decreases when DOPE is >50%, but some DOPE is required for successful folding		DOPE lateral chain pressure decreases likelihood of LacY insertion	DOPE more favourable for LacY correct folding once in the bilayer	[6]
β1-adrenergic receptor, endothelin B, GlpG, Opi3, MscL, LacY, XylE	Cell-free expression	DOPE and/or DOPG generally lead to more efficient TM insertion across the bilayer, with some exceptions that prefer DOPC or DMPC	DOPE and DOPG headgroups may have a favourable interaction with TM, increasing likelihood of TM insertion Exceptions insert efficiently enough that they do not need this early headgroup interaction to insert across the bilayer, DOPE/DOPG becomes inhibitory instead			[16–18,22,24,40] Exceptions [17,21,76]

**Figure 4.**
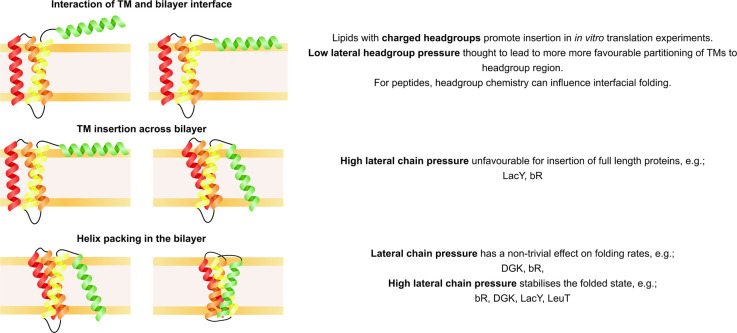
How bilayer properties affect the free energy landscape of folding. The lipid composition is known to affect insertion and folding in studies with peptides, full-length membrane proteins, and co-translationally during cell-free expression (summarised in Table 1). Here, we propose how bilayer properties may influence each stage of co-translational insertion and folding guided by such studies.

This table gives specific examples of the concepts illustrated in Figure 4. LeuT is the only example in this table which has measured specific ΔG of unfolding (ΔG_U_^H2O^) values.

From these experiments on membrane active peptides, it can be inferred that the lipid bilayer can influence one of the earliest stages in folding — the interfacial partitioning of a TM into the headgroup region of the bilayer. An optimum lipid composition will therefore ensure that the initial interaction between the TM and the bilayer is favourable. This has implications for folding *in vivo*, as some proteins may interact less favourably with the native membrane environment, and therefore be less likely to insert across the membrane without aid from the translocon, and will perhaps be more likely to misfold.

## Thermodynamic measurements of folding in the bilayer

The thermodynamics and kinetics of the folding of some full-length alpha helical membrane proteins has been measured *in vitro*. This information is usually gained by measuring the change in protein structure during unfolding by a denaturant, then measuring the refolding when the denaturant is removed (Figure 2A). These studies are performed on full-length protein which has been overexpressed in cells and purified, and there are many examples of such studies on folding in detergents [20,54–58] and bicelles [59,60]. The folding landscape will however be altered by the lipid composition that the protein is folding into (Figure 4). Lipid composition is also known to affect protein topology, the most dramatic and well-known example being the *E. coli* lactose transporter LacY [61–63]. It is known that in some cases the insertion into bilayers from a denatured state and the subsequent folding rate can be inhibited by a high lateral chain pressure (arising from using lipids such as those with PE headgroups), as it is harder to insert a fully translated protein across the membrane than it would be in lipids with a low lateral chain pressure (such as 1,2-dimyristoyl-sn-glycero-3-phosphocholine (DMPC) or 1,2-dioleoyl-sn-glycero-3-phosphocholine (DOPC)) [8,64]. Conversely, this high lateral chain pressure can lead to increased protein stability once in the bilayer [9]. In the case of LacY, 1,2-dioleoyl-*sn*-glycero-3-phosphoethanolamine (DOPE) and 1,2-dioleoyl-sn-glycero-3-phospho-(1′-rac-glycerol) (DOPG) are required for a high efficiency of reconstitution into liposomes from detergent and of refolding into liposomes from a urea-denatured state, but high DOPE becomes inhibitory [6] (Table 1).

Progress is starting to be made on measuring the free energy of folding in a lipid environment to specifically assess the effect of the lipid bilayer on thermodynamics. The bacterial leucine transporter (LeuT) is an neurotransmitter transport orthologue that is responsible for the transport of leucine in the hyperthermophilic bacteria *Aquifex aeolicus* [65]. To date, LeuT is the only alpha-helical protein to have its thermodynamic stability measured in a bilayer [5]. For these experiments, urea was used as a chemical denaturant to reversibly unfold LeuT in liposomes. By changing the lipid composition of the proteoliposomes, the effects of bilayer properties such as charge and lateral chain pressure on LeuT stability could be investigated. The stability of LeuT could be modulated by the properties of its surrounding bilayer, where addition of DOPE or DOPG to DOPC bilayers was found to increase its thermodynamic stability in liposomes [5]. This corresponds to either an increase in lateral chain pressure (DOPE) or an increase in charged headgroups (DOPG). Whilst these *in vitro* measurements are still far removed from what happens in the cell, they do allow key thermodynamic parameters in lipids to be attained. Kinetic information on LeuT folding and unfolding would further our understanding, as thermodynamic information can come from kinetic measurements of transition states and folding intermediates [7,9,14,60], and can help get a picture of the folding energy landscape and how it changes in response to different lipid environment.

## Measuring TM insertion and folding co-translationally

The remainder of this review will discuss the free energy contributions of co-translational folding and how the lipids affect TM helix insertion. Investigating the insertion of TMs and the folding of all TMs separately is useful both conceptually and experimentally but has limitations. Firstly, because folding is co-translational, folding of the initial TM helices begins while later helices are still being translated by the ribosome and inserted into the bilayer (see Figure 3). TM helices are often not independently stable and unable to insert into the bilayer by themselves [34–37]. Some helices are unable to insert into the bilayer alone, but can once other helices have been translated, or can move position or flip within the bilayer once inserted [17,62,63,66–73]. Folding studies therefore need to progress to co-translational studies in order to address how folding *in vivo* is governed. While thermodynamic data is lacking on co-translational folding, we can use previous studies on peptides and full-length proteins to make predictions of how lipids will affect the energetics of co-translational insertion.

**Figure 5.**
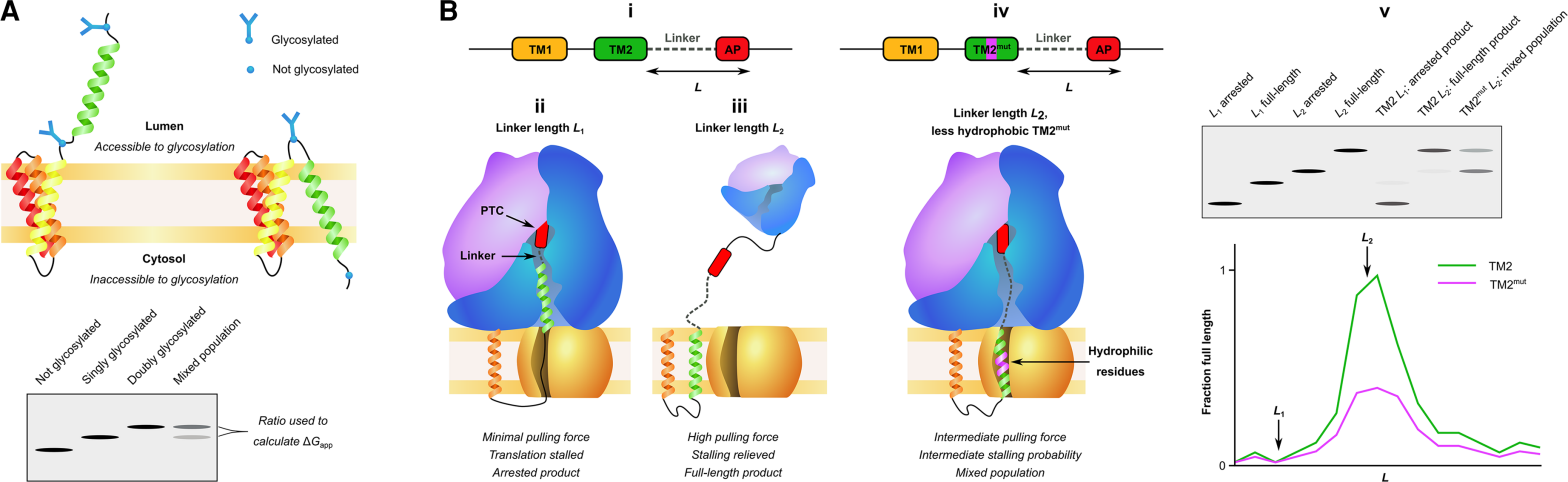
Methods to measure co-translational TM insertion *in vivo*. (**A**) Measuring TM insertion using a glycosylation assay. Glycosylation sites are engineered into the protein flanking the TM of interest. Glycosylation cannot occur on one of the sites if the TM has inserted across the membrane. The amount of single and doubly glycosylated protein is compared with calculate the ΔG_app_ of membrane insertion of that particular TM helix. (**B**) Measuring TM insertion using force profile analysis. In these experiments an arrest peptide (AP) is engineered into the protein of interest (**B**, **i**) to study the force generated by a nascent chain during translation *in vivo* [12,74,75]. APs stall translation with a duration that is proportional to the force exerted on the nascent chain during translation by the ribosome. Where little force is generated on the nascent chain (e.g. *L*_1_, where TM2 is not interacting with SecYEG, **B**, ii), the AP stalls translation and only arrested products are generated. Where a higher force is generated (e.g. *L*_2_, where TM2 is integrated into the membrane by SecYEG, **B**, iii), the AP stalling is released and a full-length product is generated. Mutations in a TM to introduce more hydrophilic residues (**B**, iv) can alter the force exerted on the nascent chain (TM2, green, compared with TM2^mut^, purple). A plot of the fraction of full-length product against the position of the AP in the nascent chain amino acid sequence (*L*) provides details into the force acting on a nascent chain during translation (**B**, v).

Early work has measured apparent free energies of TM insertion co-translationally *in vitro* by glycosylation assays (Figure 5A) [35,36], and *in vivo* using force pulling assays with stalling sequences to ascertain the force acting on TM helices during translation [12,74,75] (Figure 5B). In glycosylation assays, two glycosylation sites are engineered into the protein flanking the TM of interest. Whether the construct is singly or doubly glycosylated depends on whether the TM traversed the membrane during synthesis, and the amount of singly and doubly glycosylated protein is compared with determine the apparent free energy of membrane insertion (called ΔG_app_) [35,36]. In force pulling assays, an arrest peptide is engineered after the TM of interest to induce a stall in translation. The efficiency of stalling correlates with the efficiency of TM insertion into the membrane and is used as a proxy for the pulling force acting on the nascent chain during translation [74]. Both of these assays are powerful techniques for measuring TM insertion in native membranes, and in both the insertion efficiency of a helix can be compared either when in its native protein or on its own. The glycosylation assays were used to show that TM helices can move position once inserted, and that TM helices that cannot insert alone can do so in the full-length protein [35,36]. Force pulling assays have elucidated the contacts that form between TM helices, and between the nascent chain with the translocon during translation [12,74,75]. Both assays are however ensemble endpoint assays, so there is no information gained on the parallel folding pathways taken, or on folding kinetics. Force pulling assays have yet to be applied *in vitro* with defined synthetic membranes to investigate the specific effects of different lipids on TM insertion and folding.

**Figure 6.**
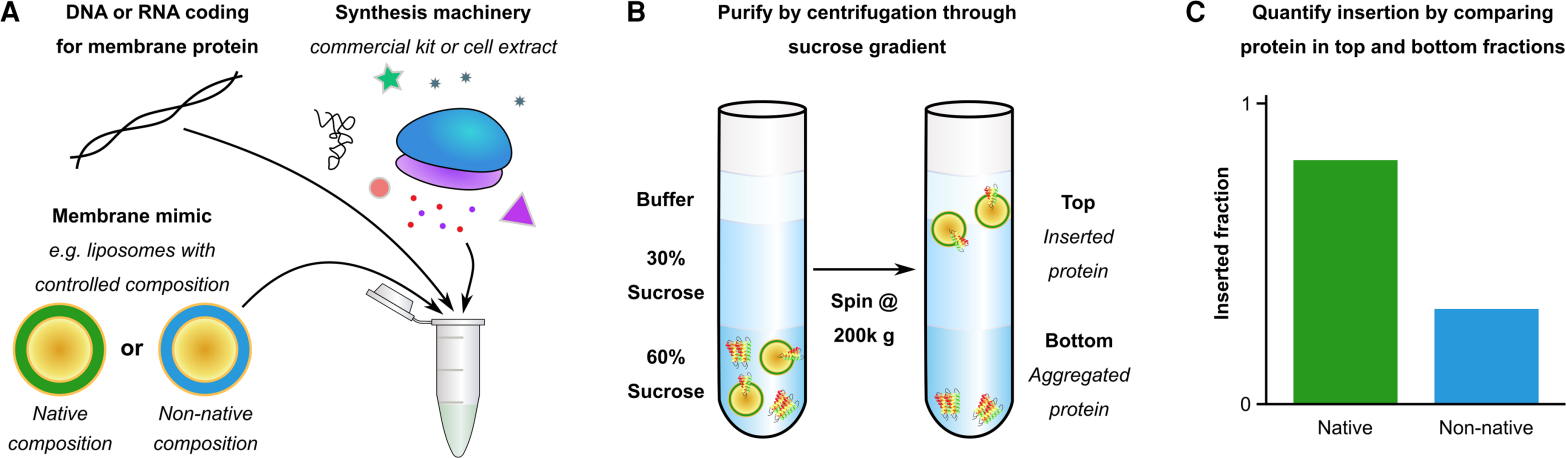
Measuring co-translational TM insertion using cell-free expression. Cell-free transcription-translation *in vitro* is used to express a membrane protein in liposomes composed of different lipids (**A**). The cell-free reaction components are separated from the liposomes on a sucrose gradient (**B**), and the total amount of protein in each lipid is quantified (by SDS–PAGE bands, radioactive counts or by the amount of functional protein) to measure the effect of lipid bilayer properties on insertion and folding (**C**). ΔG values have not yet been ascertained with this method.

Based on the peptide work and studies on full-length proteins discussed earlier, lipids are known to affect the initial TM-bilayer association. The interfacial partitioning of a TM into the headgroup region of lipids with a high lateral chain pressure, which corresponds to a low lateral headgroup pressure, is favourable as it relieves the stored curvature stress of the bilayer [8] (Figure 4). Cell-free expression systems have been used to directly study how specific changes in bilayer properties modulate co-translational TM insertion and folding (Figure 6). In comparison with *in vivo* expression, cell-free synthesis has the advantage that the composition of the bilayer structures present can be more easily controlled, and as such the influence of electrostatic and mechanical properties of the bilayer on folding can be investigated. These co-translational studies have found, with a few exceptions [17,21,76], that insertion is more favourable in lipids with charged headgroups (DOPG), or in high lateral chain pressure lipids (such as DOPE) [16–18,22,24,25,40]. This likely occurs because a charged headgroup or low lateral headgroup pressure allows favourable interfacial partitioning with the bilayer, making it more likely that a TM will insert across the bilayer and fold. Based on work on full-length proteins, it may be the case that once in the bilayer high lateral chain pressure increases folding rate and stability during co-translational folding, but this has not yet been confirmed experimentally. Interestingly, the native *E. coli* inner membrane is composed of 70% PE lipids and ∼30% PG lipids [10], corresponding to the favourable conditions found by co-translational studies. However, PE and PG lipids are not favoured by the *E. coli* protein DsbB during co-translational insertion and folding [17], implying other factors at work.

The favourable interaction of an elongating nascent chain with lipid headgroups observed *in vitro* fits neatly with a recent model of translocon-assisted insertion *in vivo*. This model states that the interfacial partitioning of the TM with the lipid headgroups is the important first step for translocon-guided insertion into the membrane [34]. For a range of peptides, transfer free energies for the spontaneous transition from interfacially bound to transmembrane state have been shown to be quantitatively similar to those measured for translocon-assisted peptide insertion [77]. This suggests that for both spontaneous and translocon-assisted insertion, the peptide transitions from an interfacially bound to transmembrane state — although presumably via different pathways [78]. It is therefore reasonable to hypothesise that the properties of the bilayer interface may affect both translocon-assisted and spontaneous insertion through similar mechanisms, and thus measurements on spontaneous insertion *in vitro* may give clues to the effects of lipids on membrane protein insertion *in vivo*. Importantly, this would suggest that the free energies measured for spontaneous insertion of short peptides, or indeed of helix-by-helix co-translational insertion of membrane proteins, would be similar to those expected for translocon-assisted insertion and therefore means that computational and different experimental techniques can complement each other, and each fill in different pieces of information.

## Conclusions

We know from previous studies on peptides and full-length proteins that lipids are very important in insertion and folding. Once a nascent chain has emerged from the ribosome exit tunnel, the lipids within the surrounding native membrane are an integral part of membrane protein folding, and therefore also directly affect the folding energy landscape. Lipids affect the initial TM-lipid interaction, where headgroup charge and low lateral headgroup pressure can promote TM association with the bilayer. Higher lateral chain pressure lipids are also known to inhibit TM insertion, but conversely also increase protein stability. We now need to understand precisely how the findings from *in vitro* studies translate to co-translational folding, in both spontaneous folding scenarios and in *in vivo* translocon-assisted folding. Some *E. coli* proteins favour the lipids found in *E. coli* inner membranes, but not all, and it is not understood why this is the case. To fully understand this, we need to build a better, higher temporal resolution understanding of co-translational folding of membrane proteins. Then we can attempt to measure the energetics of the entire system, and how the lipids affect this.

## Perspectives

Membrane proteins are important drug targets and their misfolding is implicated in many diseases. There are many different types of lipids in cellular membranes, and how these different lipids influence membrane protein folding is not well understood.
Membrane proteins interact with the interfacial region of the bilayer during translation prior to insertion across the bilayer. Lipid lateral chain pressure and headgroup charge are known to affect protein insertion, protein stability and folding rates.
Future directions should include folding studies which assess the thermodynamics and kinetics of co-translational folding in different lipids. An advance in computational methods would greatly aid these studies.

## Abbreviations

DMPC: 1,2-dimyristoyl-sn-glycero-3-phosphocholine (14:0 PC)
DOPC: 1,2-dioleoyl-sn-glycero-3-phosphocholine (18:1 (Δ9-Cis) PC)
DOPE: 1,2-dioleoyl-sn-glycero-3-phosphoethanolamine (18:1 (Δ9-Cis) PE)
DOPG: 1,2-dioleoyl-sn-glycero-3-phospho-(1′-rac-glycerol) (18:1 (Δ9-Cis) PG)
DPoPC: 1,2-dipalmitoleoyl-sn-glycero-3-phosphocholine (16:1 (Δ9-Cis) PC)
DPoPE: 1,2-dipalmitoleoyl-sn-glycero-3-phosphoethanolamine (16:1 (Δ9-Cis) PE)
HT-MD: high temperature molecular dynamics
LysoOPC: 1-oleoyl-2-hydroxy-sn-glycero-3-phosphocholine (18:1 Lyso PC)
LysoPPC: 1-palmitoyl-2-hydroxy-sn-glycero-3-phosphocholine (16:0 Lyso PC)
MD: molecular dynamics
PE: phosphatidylethanolamine
POPC: 1-palmitoyl-2-oleoyl-glycero-3-phosphocholine (16:0-18:1 PC)
POPG: 1-palmitoyl-2-oleoyl-sn-glycero-3-phospho-(1′-rac-glycerol) (16:0-18:1 PC)
SDS: sodium dodecyl Sulfate
TM: transmembrane
